# Malignant Posterior Reversible Encephalopathy Syndrome (PRES) After Allogeneic Hematopoietic Stem Cell Transplantation Requiring Emergency Decompressive Craniectomy: A Case Report

**DOI:** 10.7759/cureus.115737

**Published:** 2026-09-03

**Authors:** Rui F Gomes, Tiago O Petrucci, Miguel S Pinheiro, Pedro de Vasconcelos M, Pedro Mota, Nuno Gaibino

**Affiliations:** 1 Department of Emergency and Intensive Care Medicine, Unidade Local de Saúde Santa Maria, Lisboa, PRT; 2 University Clinic of Intensive Care Medicine, Faculdade de Medicina, Universidade de Lisboa, Lisboa, PRT; 3 Department of Neurosciences and Mental Health, Unidade Local de Saúde Santa Maria, Lisboa, PRT; 4 University Clinic of Neurosurgery, Faculdade de Medicina, Universidade de Lisboa, Lisboa, PRT; 5 Department of Oncology, Unidade Local de Saúde Santa Maria, Lisboa, PRT; 6 University Clinic of Hematology, Faculdade de Medicina, Universidade de Lisboa, Lisboa, PRT

**Keywords:** cyclosporin a, cyclosporine-induced neurotoxicity (cin), decompressive craniectomy (dc), hematopoietic stem cell transplant, myelodysplastic neoplasm with increased blasts-2, posterior reversible encephalopathy syndrome (pres), secondary intracranial hypertension

## Abstract

Posterior reversible encephalopathy syndrome (PRES) is a neurological disorder characterized by nonspecific acute neurological signs and symptoms combined with typical imaging findings. It usually has a favorable prognosis but can occasionally be complicated by severe and life-threatening manifestations. Malignant PRES is the most severe form of this condition, defined by coma, refractory intracranial hypertension, and radiological evidence of cerebral edema or hemorrhage associated with a mass effect. We report a case of a 53-year-old woman with high-risk myelodysplastic neoplasm with increased blasts-2 who underwent allogeneic hematopoietic stem cell transplantation. The post-allograft period was complicated by grade III acute graft-versus-host disease, which required intensified immunosuppression with high-dose prednisolone and therapeutic doses of cyclosporine. Eighty-three days after transplantation, the patient was admitted to the emergency department with acute neurological symptoms. Rapid development of coma and clinical signs of intracranial hypertension, refractory to osmotic therapy with mannitol and hypertonic saline, prompted an emergency decompressive craniectomy. Magnetic resonance imaging confirmed the diagnosis of malignant PRES. Withdrawal of cyclosporine, mycophenolate mofetil, and voriconazole, along with intensive supportive management in the neurocritical care unit, resulted in complete neurological recovery.

## Introduction

Posterior reversible encephalopathy syndrome (PRES) is a neurological disorder first described by Hinchey et al. in 1996 based on a case series of 15 patients [1]. It is characterized by a combination of neurological symptoms and typical brain imaging findings in the presence of various risk factors [2,3]. The most common clinical features include encephalopathy (observed in 28-94% of patients), seizures (74-87%), headache (50%), visual abnormalities (39%), and focal neurological deficits (19%). In rare cases, myelopathic signs and symptoms may be present, indicating spinal cord involvement [3].

The radiological hallmark of PRES is bilateral, symmetrical hemispheric edema on brain computed tomography (CT) or magnetic resonance imaging (MRI), which usually resolves completely on follow-up imaging. The most commonly affected structures are the parietal and occipital lobes. MRI diffusion-weighted imaging has consistently shown that these lesions represent vasogenic edema. Focal areas of restricted diffusion are uncommon and may indicate tissue infarction or cytotoxic edema. Hemorrhagic lesions are observed in approximately 15% of patients and are associated with worse outcomes [2].

The precise pathophysiological mechanism of PRES remains unknown but appears to be dependent on endothelial dysfunction. Two complementary hypotheses are usually invoked: failure of cerebral autoregulation with hyperperfusion and blood-brain barrier disruption and vasoconstriction with subsequent hypoperfusion and ischemic injury. Both hypotheses converge on endothelial damage and vasogenic edema. The early period after hematopoietic stem cell transplantation (HSCT) is particularly prone to this complication, as it combines several potential triggers, including calcineurin inhibitors (CNIs), corticosteroids, toxicity from the conditioning regimen, and systemic inflammation from infections or other complications that trigger severe inflammatory reactions [2,4].

Despite being recognized as a benign disease that usually evolves with complete resolution of neurological deficits, some patients may experience severe complications, such as intracranial hemorrhage, cerebral ischemia, hydrocephalus, brainstem compression, intracranial hypertension, and/or seizures [2,3]. Overall mortality has been observed in up to 19.0% of patients, whereas functional impairments of varying severity may persist in up to 44% of the cases [3,5]. Malignant PRES is a severe form of this condition and requires aggressive supportive care, intracranial pressure (ICP) monitoring, and immediate treatment of intracranial hypertension [6].

This article reports a case of malignant PRES developing after allogeneic HSCT. It argues for the usefulness of early decompressive craniectomy in this setting and documents the clinical and functional outcome at one year.

## Case presentation

A 53-year-old woman with a past medical history of complicated pulmonary tuberculosis, treated several years earlier with antituberculous drugs and right upper lobectomy, was diagnosed with high-risk myelodysplastic neoplasm with increased blasts-2 (MDS-IB-2; WHO 2022 criteria [7]), presenting with 18% bone marrow blasts and monoallelic TP53 mutation without 17p deletion. She had no other relevant comorbidities, in particular no history of arterial hypertension, renal disease, or autoimmune disorders. After receiving bridging hypomethylating therapy with azacitidine and a reduced-intensity conditioning regimen comprising fludarabine, melphalan, and cytarabine, with antithymocyte globulin for in vivo T-cell depletion, the patient underwent allogeneic HSCT from a matched unrelated donor (10/10 human leukocyte antigen (HLA) match).

The pre-transplant course was complicated by invasive pulmonary aspergillosis, which responded to voriconazole. Secondary antifungal prophylaxis was maintained throughout the peri-transplantation period. Bone marrow assessment on post-transplant day +47 (D+47) showed complete hematologic remission with full donor chimerism. On D+55, despite graft-versus-host disease (GVHD) prophylaxis with cyclosporine and mycophenolate mofetil, the patient developed profuse watery diarrhea (approximately 1000-1500 mL/day) and a maculopapular rash involving less than 25% of the body surface area. After infectious causes of watery diarrhea were excluded, stage 2 lower gastrointestinal GVHD and stage 1 cutaneous GVHD were diagnosed, corresponding to an overall grade III acute GVHD according to the MAGIC criteria [8]. She was treated with high-dose oral prednisolone (progressively increased to 3 mg/kg/day due to lack of clinical improvement at 2 mg/kg/day) and cyclosporine targeting blood trough levels of 200-300 ng/mL (100 mg every 12 hours, orally, with the last measured blood trough level of 342.2 ng/mL on D+64 post-HSCT). Oral mycophenolate mofetil was maintained at 1 g every 12 hours. A complete clinical response was observed on D+76, allowing tapering of the prednisolone dose to 2.5 mg/kg/day, while the cyclosporine dose was maintained.

On D+83, the patient was admitted to the emergency department after falling from her bed at night. She reported a history of progressive dizziness, gait ataxia, generalized headache, insomnia, and blurred vision over the preceding days. Initial assessment in the emergency department revealed a Glasgow Coma Scale (GCS) score of 15, severe psychomotor retardation, left-sided hemiparesis, limited levoversion of both eyes, and left-sided spatial neglect. Her initial vital signs showed arterial hypertension (162/94 mmHg), normocardia (92 beats per minute), peripheral oxygen saturation of 99% on room air, and normothermia (tympanic temperature of 36.9°C). Table 1 presents the laboratory results obtained at admission.

**Table 1:** Results of laboratory tests obtained at emergency department admission Arterial blood gas analysis was performed while the patient was breathing room air. PaCO_2_: partial pressure of carbon dioxide in arterial blood, PaO_2_: partial pressure of oxygen in arterial blood, SaO_2_: arterial oxygen saturation, MCV: mean corpuscular volume, MCH: mean corpuscular hemoglobin, MCHC: mean corpuscular hemoglobin concentration, PT: prothrombin time, aPTT: activated partial thromboplastin time, ALT: alanine aminotransferase, AST: aspartate aminotransferase, ALP: alkaline phosphatase, GGT: gamma-glutamyltransferase, LDH: lactate dehydrogenase, CK: creatine kinase, CRP: C-reactive protein, PCT: procalcitonin

Blood parameter	Patient value	Reference range
Hemoglobin	12.1	12.5-15.6 g/dL
Hematocrit	35.5	39.5-45.5%
MCV	101.8	80.0-99.0 fL
MCH	34.7	27.0-33.5 pg
MCHC	34.1	31.5-36.0 g/dL
Leukocytes	9.40	3.90-10.20 ×10⁹/L
Neutrophils	8.45	1.50-7.70 ×10⁹/L
Eosinophils	0.01	0.02-0.50 ×10⁹/L
Basophils	0.05	0.00-0.20 ×10⁹/L
Lymphocytes	0.40	1.10-4.50 ×10⁹/L
Monocytes	0.49	0.10-0.90 ×10⁹/L
Platelets	68	150-370 ×10⁹/L
PT	12.6	11.0-13.5 seconds
aPTT	33	25-35 seconds
Urea	68	16-49 mg/dL
Creatinine	1.03	0.50-0.90 mg/dL
Sodium	137	135-145 mmol/L
Potassium	3.3	3.5-5.1 mmol/L
Magnesium	1.9	1.7-2.2 mg/dL
Calcium	9.0	8.6-10.2 mg/dL
Albumin	3.8	3.5-5.2 g/dL
Glucose	97	70-110 mg/dL
ALT	566	0-33 U/L
AST	143	0-32 U/L
Total bilirubin	4.45	<1.2 mg/dL
Direct bilirubin	3.21	<0.4 mg/dL
ALP	290	44-147 U/L
GGT	1005	0-40 U/L
LDH	960	100-250 U/L
CK	48	26-192 U/L
CRP	0.06	<0.5 mg/dL
PCT	0.09	<0.5 ng/mL
pH	7.43	7.35-7.45
PaCO_2_	34.8	35.0-45.0 mmHg
PaO_2_	102	75-100 mmHg
SaO₂	97	92-99%
HCO_3_^-^	23.8	22.0-26.0
Lactate	11.0	4.5-18.0 mg/dL

An emergent head CT scan without contrast showed extensive hypodensities with a predominantly subcortical distribution involving both cerebral hemispheres in a relatively symmetric pattern, particularly in the occipital, parietal, posterior temporal, and posterior frontal regions, but with deep white matter involvement predominantly on the right side, extending to the corpus callosum and thalamus. Small acute hemorrhagic foci were observed in the left parieto-occipital region. These lesions were associated with a significant mass effect, producing sulcal effacement, ventricular compression, and cisternal obliteration, but no tonsillar herniation. Overall, the described lesions were considered compatible with PRES (Figure 1).

**Figure 1:**
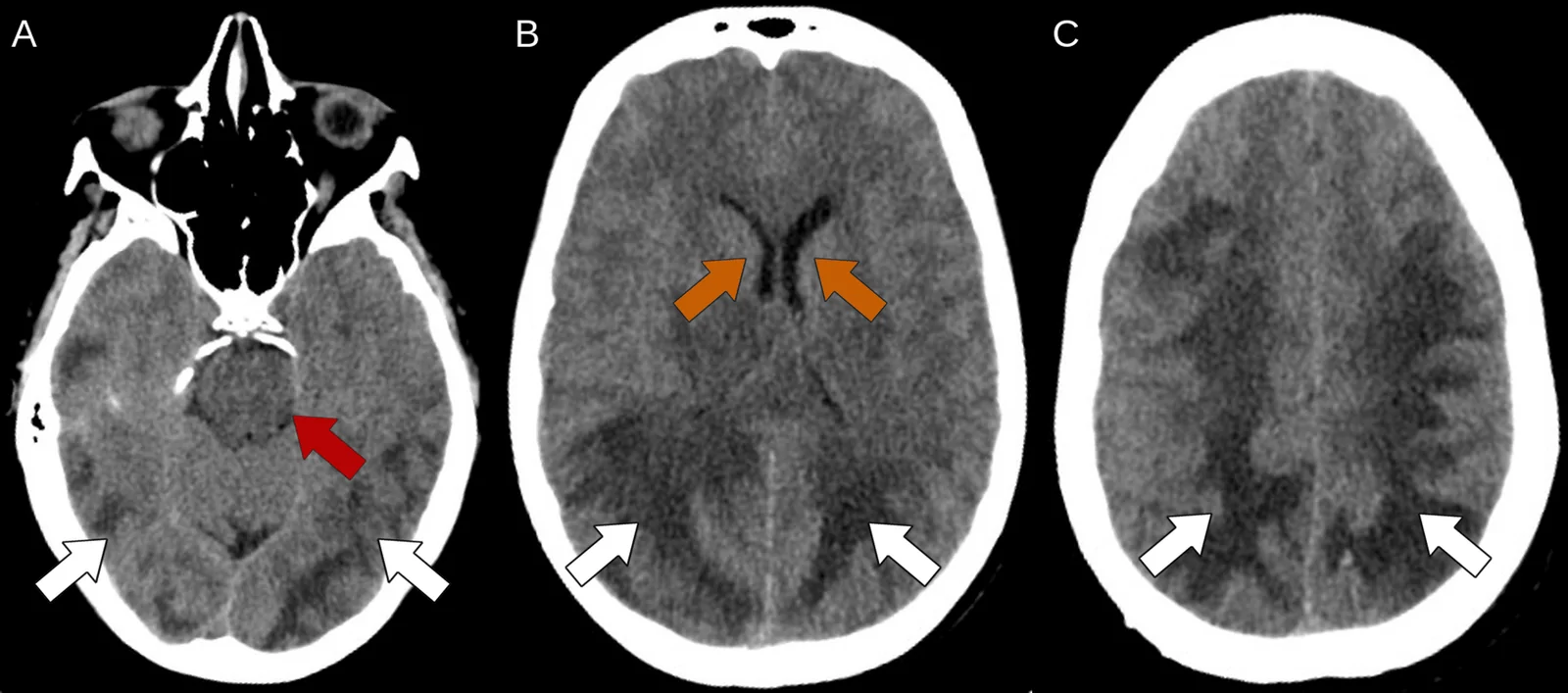
Urgent head CT scan performed upon initial observation at the emergency department Axial images from an urgent non-contrast head CT scan at three levels: (A) midbrain, (B) lateral ventricles, (C) centrum semiovale. Extensive hypodensities with a predominantly subcortical distribution involving both cerebral hemispheres in a relatively symmetric pattern, predominantly in the occipital, parietal, posterior temporal, and posterior frontal regions (white arrows). The lesions produce a marked mass effect, with diffuse sulcal effacement of both hemispheres, compression of the supratentorial ventricular system (orange arrows), and obliteration of the perimesencephalic cisterns (red arrow). The lesions were considered compatible with PRES. PRES: posterior reversible encephalopathy syndrome, CT: computed tomography

Within four hours, the patient experienced severe neurological deterioration. Her GCS score decreased to 6 (E1, V1, M4), and she developed generalized myoclonic movements, bradypnea (8 respiratory cycles per minute) with an irregular respiratory pattern, severe arterial hypertension (194/101 mmHg), and bradycardia (52 beats per minute). These clinical manifestations were consistent with Cushing’s triad, suggesting a severely increased ICP. Therefore, osmotic therapy was promptly administered (50 g of mannitol, followed by 75 mL of 3% saline), with minimal effect on the patient’s vital signs and neurological status. Endotracheal intubation was performed after induction with propofol and fentanyl to protect the airway, support ventilatory function, and reduce intracranial pressure. Controlled mechanical ventilation was initiated, and the patient remained sedated with propofol and remifentanyl infusions.

A multidisciplinary discussion involving critical care, neurosurgery, hematology, and internal medicine was immediately conducted in the emergency room. Considering the persistence of clinical signs attributable to intracranial hypertension, it was decided to proceed with an emergency right decompressive craniectomy. The decision regarding the side of decompressive craniectomy was based on the larger deep white matter involvement in the right hemisphere, producing a small leftward deviation of the midline. During the intervention, an intraparenchymal ICP sensor was placed in the right frontal lobe to allow for continuous ICP monitoring. The autologous skull bone flap was temporarily stored in an abdominal subcutaneous pocket.

After the procedure, the patient was transferred to the neurocritical care unit (Neuro-ICU). The diagnosis of PRES, along with thrombocytopenia (68,000/µL), acute kidney injury (creatinine 1.03 mg/dL in a patient with a basal creatinine level of 0.70 mg/dL), mixed liver injury (total bilirubin 4.45 mg/dL, direct bilirubin 3.21 mg/dL, alkaline phosphatase 290 U/L, gamma-glutamyltransferase 1005 U/L, alanine aminotransferase 566 U/L, aspartate aminotransferase 143 U/L), and elevated serum lactate dehydrogenase (960 U/L), was interpreted as manifestations of diffuse endothelial dysfunction. Transplant-associated thrombotic microangiopathy was considered improbable based on the absence of schistocytes on serial peripheral blood smears, persistently normal haptoglobin levels (106-172 mg/dL; normal range: 30 to 200 mg/dL), and a random urine protein-to-creatinine ratio of <1. Normal cerebrospinal fluid (CSF) analysis and negative results from both the qualitative nested multiplex PCR panel (BioFire Filmarray) and CSF culture ruled out central nervous system infection. Hepatic sinusoidal obstruction syndrome was deemed unlikely because of the absence of hepatomegaly, right upper quadrant pain, ascites, and atypical timing following conditioning chemotherapy.

Immediate management focused on withdrawing the precipitating factors of endothelial injury, controlling blood pressure (targeting a reduction of 20-25% in the first hours and a progressive decrease over the next two days, using labetalol perfusion and invasive arterial blood pressure monitoring), controlling ICP (which was persistently within the normal range after decompressive craniectomy), ensuring adequate brain perfusion pressure, and preventing secondary brain injury by controlling modifiable factors (including hypoxemia, hypo/hypercapnia, hypo/hyperglycemia, and fever). Cyclosporine was considered the most probable culprit drug and was permanently discontinued. This was based on the significantly stronger association of CNIs with PRES compared to mycophenolate mofetil or voriconazole, the temporal correlation with increased cyclosporine exposure after the diagnosis of grade III acute GVHD, and the previously documented supratherapeutic blood trough levels (342.2 ng/mL on D+64; target range, 200-300 ng/mL), despite the absence of an updated measurement at the time of PRES diagnosis. Mycophenolate mofetil and voriconazole were discontinued because of the inability to exclude a potential contribution to the neurological condition and mixed liver injury. Additionally, voriconazole is a strong CYP3A4 inhibitor that increases cyclosporine exposure. The simultaneous withdrawal of all three drugs precludes definitive attribution to a single agent.

Corticosteroid therapy was switched to intravenous methylprednisolone at an equivalent dose to ensure continued treatment of the underlying acute GVHD. In the first few hours after Neuro-ICU admission, hemorrhagic shock developed due to an acute postoperative rectus abdominis muscle hematoma caused by a high-output hemorrhage from the right inferior epigastric artery. Aggressive transfusion support was guided by rotational thromboelastometry and included platelet pools, fresh-frozen plasma, human fibrinogen concentrate, factor XIII concentrate, and packed red blood cells. The patient was readmitted to the operating room for hematoma evacuation and vascular ligation. The compromised skull bone flap was permanently disposed of.

Brain MRI was performed at the earliest opportunity, five days after decompressive craniectomy, once the patient had stabilized following the hemorrhagic shock. It revealed typical findings of PRES. Extensive bilateral subcortical abnormalities consistent with vasogenic edema were found in the frontal, parietal, and occipitotemporal regions. These abnormalities were hypointense on T1 and hyperintense on T2/FLAIR (fluid-attenuated inversion recovery), without post-contrast enhancement and with increased signal on the apparent diffusion coefficient (ADC) map. No ischemic lesions were observed. MR angiography revealed marked narrowing with irregular opacification of the bilateral anterior, middle, and posterior cerebral arteries, consistent with severe vasospasm. Postsurgical changes were also noted, including right frontoparietotemporal craniectomy with underlying linear dural enhancement and a subjacent extra-axial collection causing a mild mass effect on the adjacent brain parenchyma. The ICP sensor was correctly placed in the deep right frontal white matter of the brain. Figures 2-3 illustrate these findings.

**Figure 2:**
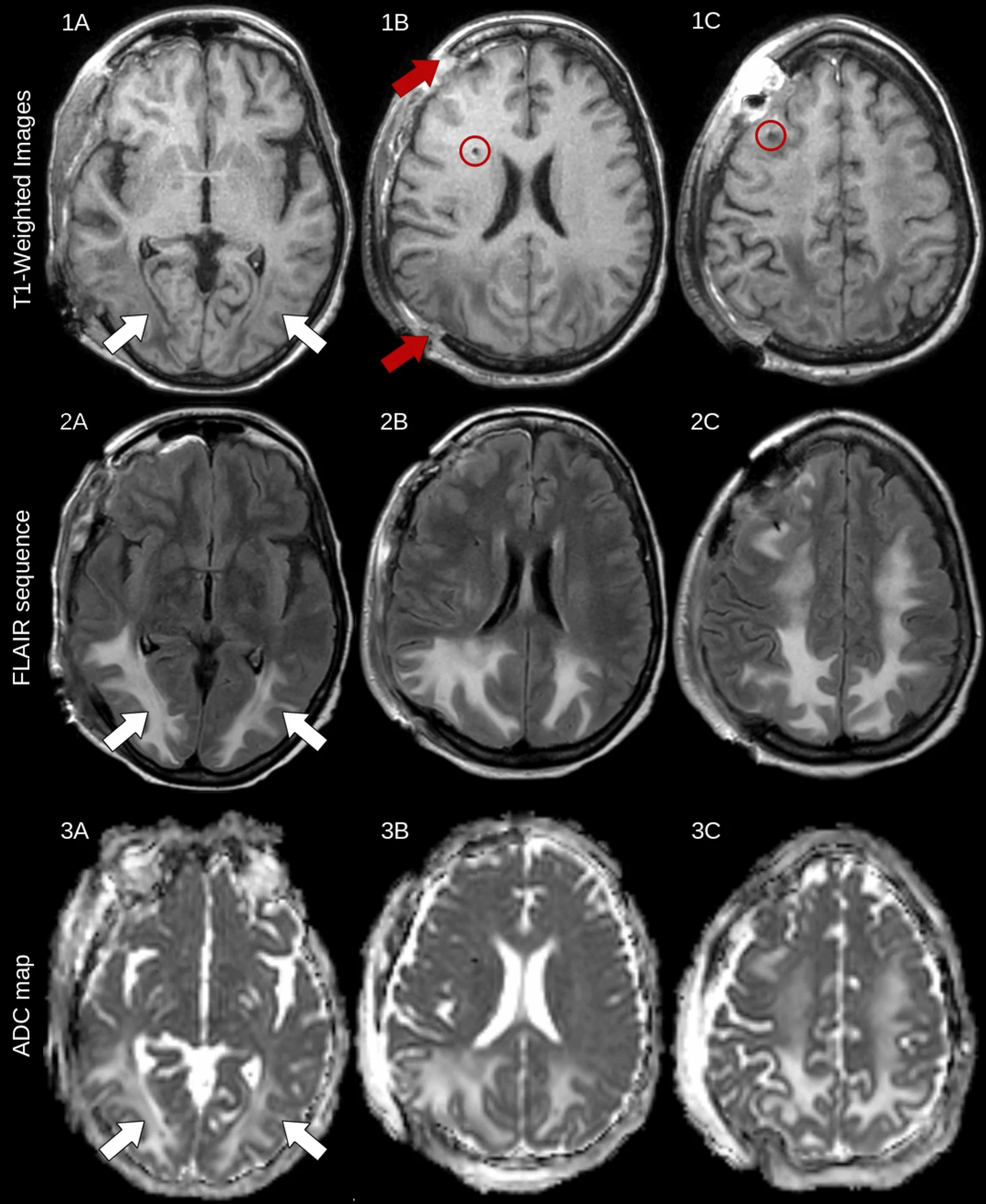
Brain MRI performed after decompressive craniectomy Axial brain MRI images were captured at three levels (from left to right: basal ganglia/occipital lobe, lateral ventricles, and centrum semiovale), showing three different sequences: T1-weighted images (1A-1C), FLAIR sequence (2A-2C), and ADC map (3A-3C). Extensive, relatively symmetric lesions of predominantly subcortical signal abnormality are seen over the high frontal and parietal convexities with bilateral occipitotemporal extension, showing hypointensity on T1 and marked hyperintensity on T2/FLAIR (white arrows on 1A and 2A; the same lesions are visible in 1B-1C and 2B-2C). The corresponding increased signal on the ADC map (white arrow on 3A) indicates facilitated diffusion, confirming vasogenic rather than cytotoxic edema, which is the hallmark of PRES. Postsurgical changes were also noted, including right frontoparietotemporal craniectomy (red arrows) with underlying linear dural enhancement and complete resolution of the midline shift. The ICP sensor was also confirmed to be correctly placed in the deep right frontal white matter (red circles). ADC: apparent diffusion coefficient, FLAIR: fluid-attenuated inversion recovery, MRI: magnetic resonance imaging, PRES: posterior reversible encephalopathy syndrome, ICP: intracranial pressure

**Figure 3:**
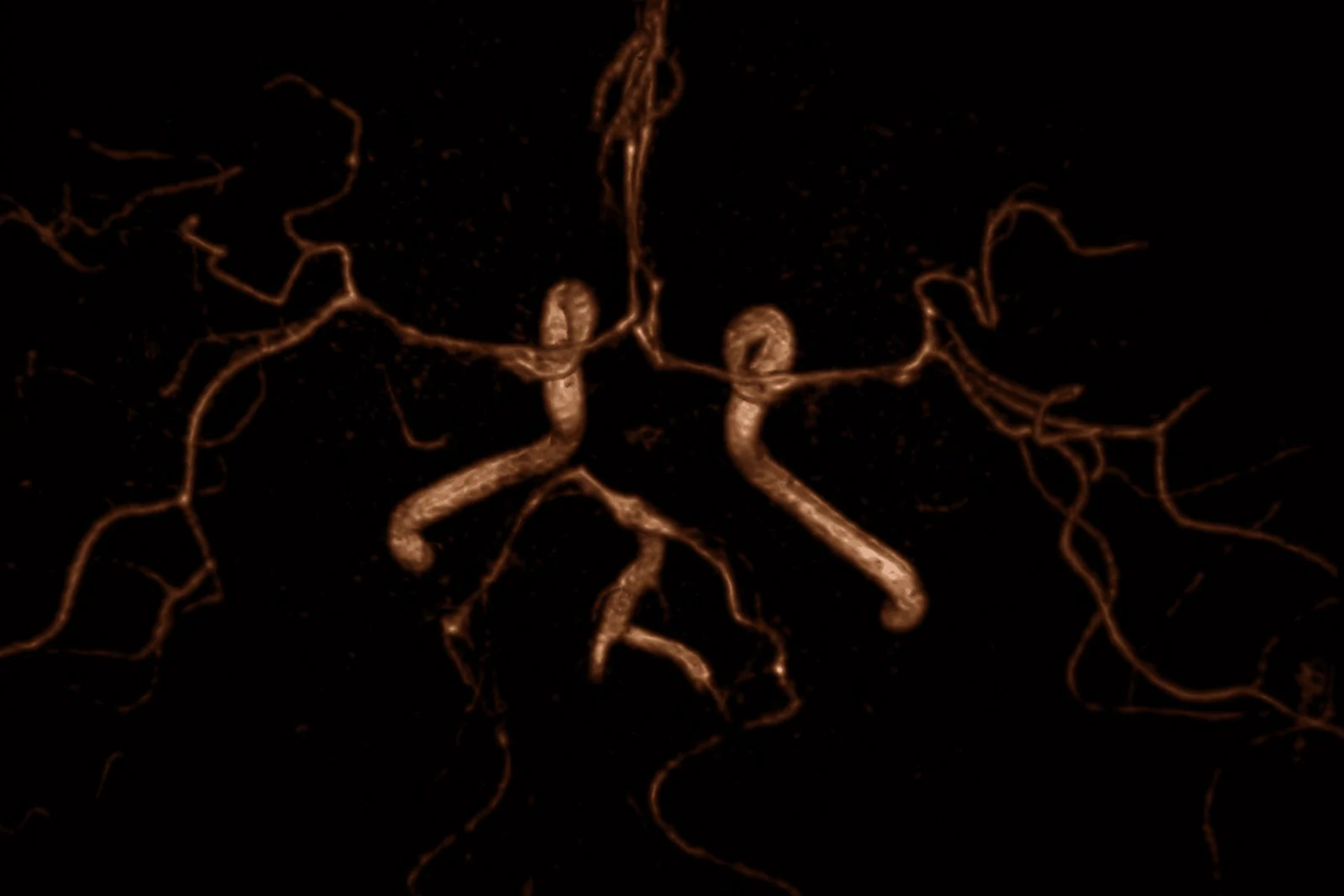
Time-of-flight MRI angiography of the circle of Willis Axial reconstruction of the TOF MRI angiography of the circle of Willis showing diffuse caliber reduction with irregular, beaded opacification of the bilateral anterior, middle, and posterior cerebral arteries, together with poor and fragmented filling of their distal cortical branches, compatible with severe diffuse vasospasm. No focal occlusion, aneurysm, or arteriovenous malformation is identified. MRI: magnetic resonance imaging, TOF: time-of-flight

Transcranial Doppler (TCD) ultrasound confirmed the presence of severe bilateral vasospasm of the anterior and middle cerebral arteries, with a maximum estimated systolic velocity of 380 cm/s, a mean velocity of 240 cm/s, and a Lindegaard Index of 9.9. Electroencephalography (EEG) demonstrated asymmetrical slow-wave background activity with active frontal and midline epileptiform discharges. Considering these changes, antiepileptic therapy was initiated with intravenous levetiracetam (1.5 g every 12 hours). Therapy was subsequently augmented with clonazepam (1 mg every eight hours) due to the persistence of epileptiform activity on EEG. Additionally, empiric off-label enteric nimodipine (60 mg every four hours) was administered to address severe vasospasm, following a multidisciplinary consensus involving neurology and neurocritical care specialists. There was no significant effect on blood pressure control.

After stabilization of the neuromonitoring parameters with adequately controlled ICP (12-16 mmHg), resolution of severe vasospasm, normalization of the EEG pattern, and exclusion of ischemic and hemorrhagic complications, sedation was gradually weaned. The intraparenchymal ICP sensor was removed 9 days after decompressive craniectomy without complications. Full recovery of consciousness was documented, allowing for a spontaneous breathing trial and subsequent extubation on the 11th day of Neuro-ICU stay. Post-extubation neurological assessment showed full recovery from the focal neurological deficits present upon hospital admission. However, symmetrical flaccid tetraparesis with preserved sensory function was documented, consistent with intensive care unit-acquired weakness, with a Medical Research Council sum score of 28. Along with neurological improvement, laboratory tests showed complete resolution of mixed liver injury and acute kidney injury.

Following enhanced rehabilitation, the patient was transferred to the hematology department on the 16th day of Neuro-ICU stay and D+98 after the HSCT. During her stay in the hematology department, intensive physical, pulmonary, and neurological rehabilitation was maintained, leading to the complete resolution of physical impairments. However, persistent dysphagia and recurrent episodes of pneumonia led to complementary investigations using bronchofibroscopy and upper gastrointestinal endoscopy. A tracheoesophageal fistula located 3 cm below the vocal cords was diagnosed, which was most probably secondary to endotracheal intubation. Tracheal stenting was considered but was technically infeasible due to the fistula's high location. For this reason, surgical correction was planned, but the patient showed favorable clinical improvement with spontaneous closure of the tracheoesophageal fistula following conservative management with tube feeding. This complication prolonged hospitalization, culminating in the patient's discharge on the 118th day following hospital admission. Table 2 presents a concise timeline of major clinical events.

**Table 2:** Chronology of major clinical events ADC: apparent diffusion coefficient, CT: computed tomography, ED: emergency department, EEG: electroencephalography, FLAIR: fluid-attenuated inversion recovery, GCS: Glasgow Coma Scale, GOS-E: Glasgow Outcome Scale-Extended, GVHD: graft-versus-host disease, HLA: human leukocyte antigen, HSCT: hematopoietic stem cell transplantation, ICP: intracranial pressure, ICU: intensive care unit, IV: intravenous, MDS-IB-2: myelodysplastic neoplasm with increased blasts-2, MRC: Medical Research Council, MRI: magnetic resonance imaging, mRS: modified Rankin Scale, Neuro-ICU: neurocritical care unit, PRES: posterior reversible encephalopathy syndrome, TCD: transcranial Doppler

Time point	Event	Key details
Pre-transplant	High-risk MDS-IB-2 (18% bone marrow blasts)	Azacitidine. Invasive pulmonary aspergillosis treated with voriconazole.
Day 0	Allogeneic HSCT	10/10 HLA-matched unrelated donor. Reduced-intensity conditioning regimen (fludarabine, melphalan, cytarabine). GVHD prophylaxis with cyclosporine, mycophenolate mofetil, and T-cell depletion with antithymocyte globulin.
D+47	Complete hematologic remission	Full donor chimerism.
D+55	Grade III acute GVHD	Increased doses of prednisolone (up to 3 mg/kg/day) and cyclosporine (target trough levels: 200-300 ng/mL). Mycophenolate mofetil dose maintained (1 g every 12 hours).
D+76	GVHD complete response	Prednisolone tapering. Cyclosporine dose maintained.
D+83	ED admission because of neurological symptoms	Fall from the bed, dizziness, gait ataxia, generalized headache, insomnia, and blurred vision. At hospital admission: GCS 15, severe psychomotor retardation, left-sided hemiparesis, limited levoversion of both eyes, left-sided spatial neglect, arterial hypertension (162/94 mmHg). Urgent head CT scan: extensive symmetrical subcortical hypodensities associated with marked mass effect, compatible with PRES (Figure 1).
D+83, a few hours later	Refractory intracranial hypertension	GCS 6, Cushing's triad and generalized myoclonic movements. Ineffective mannitol therapy → endotracheal intubation, emergency decompressive craniectomy and ICP sensor placement.
D+83, Neuro-ICU day 1	Culprit drug withdrawal, control of arterial hypertension	Cyclosporine and MMF stopped. Steroid therapy switched to IV methylprednisolone.
D+84, Neuro-ICU day 2	Hemorrhagic shock	Acute postoperative rectus abdominis muscle hematoma due to right inferior epigastric artery bleeding → Urgent surgical revision: hematoma evacuation, vascular ligation, and surgical disposal of the compromised skull bone flap.
Neuro-ICU first days	PRES confirmed by MRI. Development of severe vasospasm and seizures	Brain MRI: vasogenic edema with T2/FLAIR hyperintensity and increased signal in the ADC map (Figures 2-3). TCD: severe bilateral vasospasm of the anterior and middle cerebral arteries (Lindegaard 9.9). EEG: asymmetrical slow-wave background activity with active frontal and midline epileptiform discharges → nimodipine, levetiracetam, clonazepam
D+91, Neuro-ICU day 9	ICP sensor removal	ICP 12-16 mmHg, vasospasm resolved, EEG normalized. No ischemic or hemorrhagic complications on brain CT scan.
D+93, Neuro-ICU day 11	Extubation	Resolution of the focal neurological deficits. ICU-acquired weakness (MRC sum score 28).
D+98, Neuro-ICU day 16	Transfer to hematology	Enhanced physical rehabilitation with full recovery of the physical impairments.
Hematology ward	Tracheoesophageal fistula	Post-intubation complication: tracheoesophageal fistula 3 cm below the vocal cords → stenting unfeasible, spontaneous closure while waiting for surgical repair.
D+118	Hospital discharge	Favorable outcome with full recovery from the neurological deficits: mRS 1, GOSE 8.

After discharge, the patient enrolled in a structured follow-up program at our hospital. This included hematology consultations one week after discharge and at least monthly over the following year. Additionally, infectious disease consultations were conducted due to the pre-transplant diagnosis of pulmonary aspergillosis and the patient’s immunosuppressed state. Thoracic surgery consultations were scheduled at one and two months post-discharge.

The patient achieved complete hematologic remission for MDS-IB-2 after HSCT, with sustained remission documented over 20 months of follow-up. Neither GVHD nor the tracheoesophageal fistula recurred during follow-up. The patient was offered delayed alloplastic cranioplasty of the right frontoparietotemporal defect but refused the procedure. After a one-year follow-up, the patient maintained full neurological recovery, as indicated by a Glasgow Outcome Scale-Extended score of 8 and a modified Rankin Scale score of 1, with only mild headache and mood changes reported.

## Discussion

The precise pathophysiological mechanism of PRES remains unclear and may differ across its forms. The most widely accepted hypothesis suggests that hypertensive episodes that exceed the upper limit of cerebral blood flow autoregulation may lead to hyperperfusion, causing endothelial cell damage and disruption of the blood-brain barrier. Regardless of the initial trigger, endothelial dysfunction is crucial to the underlying pathophysiology. In fact, in 20% of patients without arterial hypertension, endothelial dysfunction caused by toxic drugs or increased cytokine levels seems to be the driving mechanism for PRES development [5]. HSCT is a well-recognized risk factor for PRES. Its incidence after bone marrow transplantation with reduced-intensity conditioning and cyclosporine immunosuppression is approximately 7-9% and appears higher (16%) after myeloablative regimens [2]. Several PRES-related risks coexist after transplantation. One of the most frequently described risk factors/triggers in the literature is immunosuppressive therapy with CNIs. Cyclosporine and tacrolimus are the most well-studied CNIs [9]. These drugs induce endothelial activation and inflammatory cytokine production through the TLR4/MyD88/NF-κB and TLR4/TRIF/NF-κB signaling pathways and the generation of reactive oxygen species in endothelial and vascular smooth muscle cells, leading to endothelial cell apoptosis, enhanced oxidative stress, and inflammation [10]. Moreover, CNIs affect vascular function by inhibiting nitric oxide production, thereby impairing endothelium-dependent vasodilation and promoting vasoconstriction. The resulting increase in systemic vascular resistance significantly contributes to the development of arterial hypertension [11]. Together, endothelial dysfunction/injury, cerebral vasoconstriction, and systemic arterial hypertension may lead to altered blood-brain barrier integrity and consequent vasogenic edema, as is usually seen in PRES [2,4]. The reason why some similarly treated patients develop this complication while others do not is currently poorly understood, but additional insults to pre-existing endothelial cell injury and endothelial vulnerability may cross the threshold for clinically significant endothelial injury [4,5,9].

PRES typically develops in the early post-transplantation period, usually between one week and three months after allografting [12]. This was the case for our patient, who developed the first symptoms around D+80 after HSCT. It specifically occurred following the diagnosis of grade III acute GVHD, which was managed with high-dose prednisolone and an increased dose of cyclosporine. The association between acute GVHD and the occurrence of PRES following HSCT has been reported in the literature [13,14] and may be driven by the intensity of the conditioning regimen used, the inflammatory state associated with acute GVHD, and the higher doses of CNIs and steroids used to treat this complication, leading to diffuse endothelial activation and widespread injury to the central nervous system [13].

Discontinuation of CNIs in the face of severe neurotoxicity typically leads to symptom reversal and resolution of toxicity [14]. Evidence for CNI-induced PRES after solid organ transplantation shows that neurological signs usually resolve within a few days (median of 6 days). Still, neuroimaging abnormalities generally require a longer period (median of 30 days) [15]. However, evidence regarding symptom duration and neuroimaging abnormalities in the specific context of HSCT remains limited.

Nonetheless, some patients may experience severe forms of PRES that can be fatal or lead to persistent neurological impairment. Recurrence of neurological symptoms is also reported in almost 4% of patients [16]. It has been associated with sickle cell disease, autoimmune conditions, hypertensive crisis, renal failure, multiorgan failure, and HSCT complicated by infection [2,3]. Malignant PRES is a severe form of the disease, as defined by Akins et al. based on a case series of five patients using the following three criteria: (1) GCS score < 8; (2) clinical deterioration despite standard medical treatment for elevated ICP; and (3) radiographic evidence of cerebral edema or hemorrhage exerting a mass effect, effacement of the basal cisterns, and/or transtentorial, tonsillar, or uncal herniation [6]. Failure of medical treatment in controlling elevated ICP in this clinical context is not unexpected, as malignant PRES contributes to an increase in the intracranial vault's content through extensive edema and, sometimes, intracranial hemorrhage. In this context, aggressive management of associated conditions, such as severe arterial hypertension and coagulopathy, early consideration of craniectomy and evacuation of confluent hemorrhages, and multidisciplinary management with neurocritical care and neurosurgery guidance should be pursued in all patients who meet the criteria for malignant PRES [6].

Despite the severe clinical presentation of malignant PRES, with early recognition, timely management of precipitating factors, and aggressive treatment, complete reversal of neurological symptoms is possible, as observed in our patient. Overall, the long-term outcomes among surviving patients are usually favorable, with a reported modified Rankin Scale score of ≤2 at one year after hospital discharge in almost all cases [6,16]. The subset of patients who usually have worse outcomes comprises those with hemorrhagic PRES, with reported mortality rates of up to 29% [17,18]. An increased risk of hemorrhagic PRES has been associated with coagulopathy, anticoagulation, HSCT, solid organ transplantation, systemic lupus erythematosus, pregnancy, immunosuppression, and chemotherapy [6].

The pathophysiological mechanisms described above for CNI-induced neurotoxicity may also underlie other acute organ dysfunctions, namely acute kidney and liver injury [19,20]. Nephrotoxicity is one of the most common adverse effects of CNIs in transplantation and results from a combination of vascular, tubular, and functional toxicity in a dose-dependent manner [20]. Hepatotoxicity is less frequent and usually mild, typically presenting as elevated serum bilirubin without significant aminotransferase changes, and is mainly driven by inhibition of the bile salt export pump. Recovery after cyclosporine withdrawal is generally rapid [19]. In our patient, both dysfunctions were present at emergency department admission and completely reversed after drug withdrawal, without other specific interventions or identification of alternative diagnoses, supporting systemic CNI toxicity.

## Conclusions

PRES should always be included in the differential diagnosis of acute neurological symptoms in HSCT recipients, given its nonspecific presentation and multiple risk factors during the post-allograft period. The risk appears to be higher in the first months after HSCT and in patients experiencing complications, such as GVHD and infections. Immunosuppression with CNIs is one of the most frequent causes of PRES in this population, mainly driven by diffuse endothelial dysfunction, which may also induce other acute organ dysfunctions, such as acute kidney injury and mixed liver injury.

This case report highlights four clinically relevant aspects: (1) the differential diagnosis of acute neurological symptoms in the post-HSCT period is extensive, encompassing infections, drug-induced neurotoxicity, transplant-associated thrombotic microangiopathy, hematologic malignancy involvement of the central nervous system, ischemic stroke, intracranial hemorrhage, and others; (2) HSCT involves multiple risk factors whose cumulative effects may trigger PRES; (3) early aggressive multidisciplinary management, including withdrawal of the precipitating drugs, neurocritical care, and early decompressive craniectomy, contributed to survival and favorable recovery in this patient; and (4) despite the severe clinical presentation of malignant PRES, complete neurological recovery is possible. A single case report cannot establish decompressive craniectomy as standard treatment for malignant PRES, nor define patient selection or timing for surgery. However, it suggests that early craniectomy deserves multidisciplinary consideration when intracranial hypertension fails to respond to medical management.
